# Preventative Care in First Responder Mental Health: Focusing on Access and Utilization *via* Stepped Telehealth Care

**DOI:** 10.3389/frhs.2022.848138

**Published:** 2022-06-09

**Authors:** Hannah M. Wright, Dianna Fuessel-Hermann, Myah Pazdera, Somi Lee, Brook Ridge, Joseph U. Kim, Kelly Konopacki, Layne Hilton, Michael Greensides, Scott A. Langenecker, Andrew J. Smith

**Affiliations:** ^1^Department of Psychiatry, University of Utah School of Medicine, Huntsman Mental Health Institute, Salt Lake City, UT, United States; ^2^Salt Lake City Veterans Affairs (VA) Medical Center, Salt Lake City, UT, United States; ^3^United Fire Authority, Salt Lake City, UT, United States; ^4^Department of Psychiatry, Geisel School of Medicine at Dartmouth, Hanover, NH, United States; ^5^Lyda Hill Institute for Human Resilience, University of Colorado, Colorado Springs, CO, United States

**Keywords:** occupational stress, utilization, preventative medicine, first responders, PTSD, depression, alcohol, stepped care

## Abstract

First responders are at high risk for disorders that arise from repeat exposure to stress and trauma (Post Traumatic Stress Disorder, depression, and problematic alcohol use). Although mental health treatments are available, first responders often do not access them, anchored by barriers that include: lack of knowledge, stigma, negative experience with mental health providers, and time-based burdens. In this study, we designed an intervention to address these barriers, extending a Planned-Action framework. Step 1 involved self-report screening for four mental health risks (PTSD, depression, anxiety, and alcohol use risk), delivered to all personnel electronically, who were free to either consent and participate or opt-out. The detection of risk(s) in Step 1 led to scheduling a Step 2 telehealth appointment with a trained clinician. We report descriptive statistics for participation/attrition/utilization in Steps 1 and 2, rates of risk on four mental health variables, and rate of adherence to follow-up treatment recommendations. Step 1: In total, 53.3% of personnel [229 of 429 full-time employees (221 males; eight females; 95% White; 48% paramedic or Emergency Medical Technician; 25% captain; 19% engineer; 7% other)] initially opted-in by consenting and completing the brief remote screening survey. Among those who opted-in and completed (*n* = 229), 43% screened positive for one or more of the following mental health risks: PTSD (7.9%); depression (9.6%); anxiety (13.5%); alcohol use (36.7%). Step 2: A maximum of three attempts were made to schedule “at risk” individuals into Step 2 (*n* = 99). Among the 99 who demonstrated a need for mental health treatment (by screening positive for one or more risk), 56 (56.6%) engaged in the telehealth appointment. Of the 56 who participated in Step 2 clinical appointments, 38 were recommended for further intervention (16.6% of full-time personnel who participated). Among the 38 firefighters who were recommended to seek further mental health services, 29 were adherent/followed through (76.3% of those who received recommendations for further services). Taken together, evidence-based, culturally conscious, stepped care models delivered *via* the virtual/telehealth medium can promote access, utilization, and cost-effective mental health services for first responders. Implications are for informing larger, more rigorous dissemination and implementation efforts.

## Introduction

Serving as a firefighter involves chronic stress and trauma exposures incurred during occupational events such as structure fires, wildfires, traffic accidents, suicides, drug overdoses, Sudden Infant Death Syndrome, domestic violence scenes, and disasters. Firefighters work long hours under shift work schedule demands and mandatory staffing, prompting deterioration of natural coping resources [e.g., social supports, restorative sleep; (1–4)]. Chronic physical and psychological stress exposures, paired with degradation of natural coping processes, leaves firefighters at risk for physical and mental health problems [e.g., (5–14)]. A recent study showed that more than 50% of firefighters may be at risk for PTSD, depression, anxiety, and/or alcohol use disorder (15). This is a population in need of culturally tailored, preventative, accessible treatment resources to facilitate mental health and wellbeing.

Evidence-based treatments are available for the common types of mental health problems that first responders are at risk for, including treatments for PTSD (Prolonged Exposure, Cognitive Processing, Eye Movement Desensitization and Reprocessing) and depression (Cognitive Behavioral Therapy Treatment approaches) (16–21). Notwithstanding the efficacy of evidence-based mental health treatments [e.g., see (22)], fewer than half of first responders in need of mental health care seek treatment (23). A recent survey of nearly 40,000 firefighters revealed that among firefighters with a probable PTSD diagnosis, fewer than 10% had sought treatment in the past month (24).

Reasons for not seeking mental health treatments are undoubtedly complex. For example, emergency responder cultures value archetypes of self-reliance, self-sufficiency, playing heroic roles, and saving other people at risk to the self (25). Firefighters are often unaware of the potential mental health risks for long-term effects of persistent stress (26), and/or lack the knowledge for where and how to access mental health providers trained in evidence-based approaches (27). Among firefighters who are identified as needing mental health services, seeking such services can be blocked by stigma and fear of negative professional consequences, losses of standing, promotion potential, and/or pay (10, 26, 28). Additionally, practical barriers exist for being able to attend health appointments consistently due to shift work, mandatory staffing practices, and associated time constraints (24, 27).

A recent community-based study identified four critical facilitators for improving mental health service use among first responders: increasing knowledge, reducing stigma, increasing positive experience with mental health providers, and removing time-based burdens (27). This study by Jones et al. (27) addressed initial steps in a Planned-Action based framework for translating research into mental health care practice for first responders having (step 1) identified the problem (i.e., first responders underutilizing mental health care), (steps 2 and 3) reviewed the evidence and adaptee toward innovation, and (step 4) assessed barriers to uptake [see (29, 30)]. The first responder community would benefit from interventions that translate this knowledge into practice.

In the current study, we sought to extend the next three steps in a Planned-Action framework by (a) developing an intervention that applies/translates known barriers and facilitators (27), (b) implementing the intervention, and (c) evaluating uptake and utilization (29, 30). Within the Planned-Action framework, we incorporated a rational stepped care design feature to optimize for cost-effectiveness, future scalability, and increased access in a scarce resource context (31–38). The overarching goal of the current manuscript is to describe the intervention development, implementation, and utilization process as a means to improve first responder mental health.

## Materials and Methods

### Design and Setting

The University of Utah Institutional Review Board approved all study procedures before the initiation of the intervention. Attempts were made to contact all full-time personnel (with no exclusions) in a fire department in the Rocky Mountain West (*n* = 429) to participate in this intervention. An administrator at the first responder agency provided the clinical team with a personnel list including names and contact information for every first responder in the department. The clinical team generated a unique, secure survey link associated with each member of the department (generated using REDCap), and the list of first responder names and unique survey links (containing access to the Step 1 intervention survey described below) was provided back to the mental health liaison at the first responder agency. The agency liaison sent the unique Step 1 survey links *via* text message, a participation maximizing effort, that the survey link be received from “within tribe” (i.e., from a member of the first responder department). Once they received the survey link, participation required agreeing to informed consent. Informed consent provided information about the aim of the survey, data collection and storage procedures, as well as potential risks and benefits of participation (e.g., feedback about wellbeing, access to mental health professionals, consultation). When agreeing to informed consent, participants were agreeing that if they screened positive for any mental health risk, they would be contacted by a scheduling assistant from the clinical team to schedule an appointment with a mental health provider. Individuals who agreed/consented were directed to a brief battery of screening questions (completion time ~1 min). Individuals who declined consent were provided a list of mental health resources and discontinued from participation. Further details of the intervention are described below.

**Figure 1:**
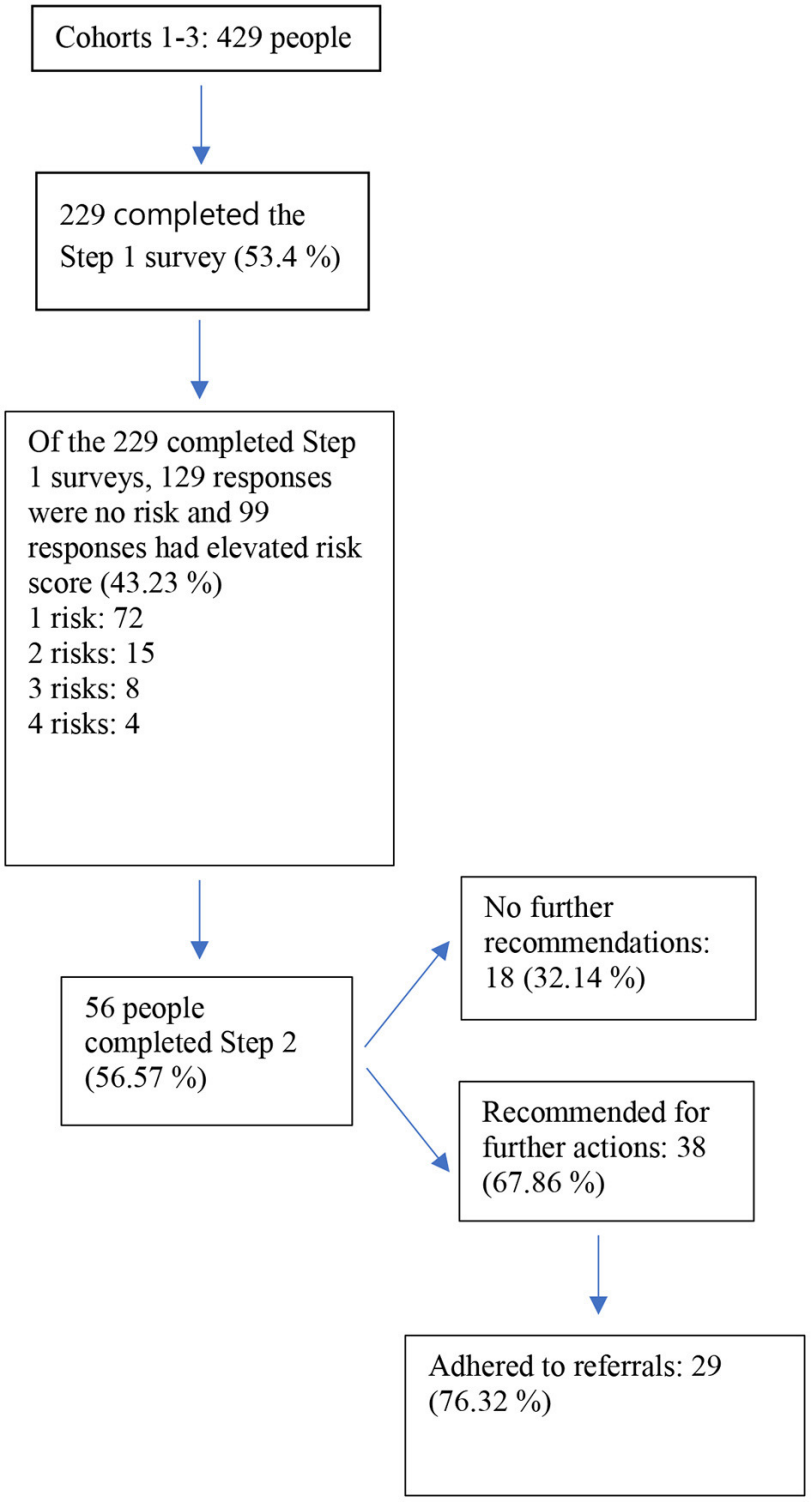
Participation in each step.

A total of 229 individuals (roughly 53% of the department) participated (96.5% male, 95.2% White, mean age = 42.28 [SD = 9.64]), including paramedics (47.8%), captains (24.6%), engineers or drivers (19.3%), chiefs (5.3%), administration (2.2%), or other (0.9%). The current sample is comparable to national firefighter demographics (39). See Figure 1 for a summary of participation by each step in the model.

### Overview of Development and Pilot Implementation

Improving mental health treatment access and utilization has been the focus of dissemination and implementation science for several decades (40). Whereas, some barriers and facilitators to treatment are universal (e.g., availability of trained providers), others are specific to populations [see (27)]. We sought to develop an intervention that would improve access and utilization by addressing first responder specific barriers and facilitators to mental health services. To do so, we used a Process-Based framework [i.e., Planned-Action; (29, 30)] to extend the evidence elucidated by Jones et al. (27). As such, our intervention design choices were as follows:

(1) Maximize flexibility and reduce time-based burdens/constraints by (a) using a brief risk screening instrument that reduces participant burden and (b) offering appointments *via* virtual telehealth that allowed for first responders to schedule at their convenience.
(2) Reduce stigma by (a) delivering this as an intervention that first responders in the participating agency could “opt out” of and (b) communicate/conceptualize mental health distress through an occupational stress/performance enhancement framework.
(3) Increase knowledge by incorporating mental health education and introducing skills aimed at enhancing mental health.
(4) Increase positive experiences with mental health providers by (a) training providers on first responder language and culture and (b) communicating/conceptualizing mental health distress through a culturally palatable (i.e., in first responder cultures) occupational stress/performance enhancement framework.

Additionally, we addressed rational considerations for cost and efficiency of distributing therapeutic resources within healthcare systems using a stepped care design (32–34, 36, 37). Stepped care involves providing treatment to patients in the least restrictive setting while continuously monitoring the effectiveness of each “step” in the treatment model (31). Moreover, stepped care models may have complimentary value for improving access by tailoring the treatment amount and type to the level of care needed (31, 38) which in turn optimizes the financial and time cost to both patient and clinician (38) and allows for efficiency of utilizing scarce resources (35). Finally, a stepped care approach has scalability implications by aiming to operationalize a set of reproducible, standardized procedures (37, 41), often complemented by the use of technology (42).

### Step 1

All 429 personnel received a text message including a secure survey link unique to that person from a mental health liaison in their organization. Upon agreeing to informed consent, individuals completed a self-report questionnaire (completion length ~1 min) comprised of 19 questions: six demographic questions [gender, race/ethnicity, career length, recruit/new hire status (yes/no), and primary occupational role]; and 13 questions screening for trauma history and symptoms of traumatic stress, depression, anxiety, and alcohol use. Participants who declined informed consent were provided with a list of mental health service contacts for therapy and/or crisis services and discontinued from the intervention. Engagement with the survey was monitored by the clinical team, and if participants did not engage [i.e., did not open the survey or complete consent (either agree or disagree)], up to three attempts were made to contact them before discontinuing them in the program.

Following completion of the Step 1 screening survey, participants who screened as “positive” on one or more of the measures (traumatic stress, depression, anxiety, alcohol use) were contacted within 48–72 h. Participants who screened positive for mental health risk(s) and who were contacted successfully were offered the opportunity to have a 60-min appointment with a mental health professional (Step 2). Notably, some participants screened positive for mental health risks and were successfully contacted in attempts to schedule for Step 2 virtual clinical appointment, but chose to decline continued participation in the service. Individuals who did not screen positive for a mental health risk received a text message indicating that no risk was identified at this time and were provided with contacts for mental health services and crisis services should such a mental health need exist nonetheless.

#### Step 1 Measures

##### Traumatic Stress Symptoms

Traumatic stress was assessed with an adapted version of the Primary Care PTSD Screen for DSM-5 [PC-PTSD-5; (43)] consisting of five questions used to reflect the Likert scale associated with the PCL-5 [0 = not at all, 1 = a little bit, 2 = moderately, 3 = quite a bit, 4 = extremely; see (44–46)]. The five items were summed together to obtain a continuous total score (range = 0–20) with of 10 or above indicating at risk. Internal consistency was high for this sample (Chronbach's α = 0.85).

##### Depression

The Patient Health Questionnaire-2 [PHQ-2; (47)] is a brief depression screener consisting of two items answered on a 4-point Likert scale (0 = not at all to 3 = nearly every day). Items were summed with a total continuous score of 3 or above used to indicate risk (47). Internal consistency was adequate for this sample (Chronbach's α = 0.81).

##### Anxiety

The Generalized Anxiety Disorder Scale-2 (48) is a brief two-item screener of anxiety symptoms answered on a 4-point Likert scale (0 = not at all to 3 = nearly every day). Items were summed with a total continuous score of 3 or above used to indicate risk. Internal consistency was low for this sample (Chronbach's α = 0.50).

##### Alcohol Use

The Alcohol Use Disorders Identification Test-Consumption Questions [AUDIT-C; (49)] contains three items measuring alcohol use frequency and quantity. Items were summed with a total score of 4 for men and 3 for women indicating risk. Internal consistency was adequate for this sample (Chronbach's α = 0.70).

### Step 2

Before engagement in the virtual clinical interview (Step 2), participants who screened positive in Step 1 and agreed to schedule a Step 2 appointment were sent a new survey link, which was automated to be sent 1 h before the appointment with instructions to complete prior to the interview. The Step 2 survey represented an expanded version of Step 1 with a focus on more in-depth, reliable measures of PTSD (PCL-5; Weathers et al., 2012) and depression [PHQ-9; (50)] to provide clinicians with more clinically actionable information (e.g., which dimensions of PTSD and mood were most clinically prominent). During the scheduled appointment participants engaged in a 60-min clinical interview with a mental health provider (psychologists or licensed clinical social workers) comprised of four aspects.

(1) Psychosocial and functional assessment (~30 min).
(2) Interactive education about the relationship between chronic stress and mental/physical health tailored to the problems identified in the assessment (~15 min).
(3) Introduction of a brief problem-focused coping skill (~10 min).
(4) Recommendation/referral. After the clinical interview possible referrals were made as follows: (a) trauma/PTSD focused therapy; (b) brief neurocognitive screening (Step 3 in the intervention); (c) medication evaluation; (d) behavioral health interventions; (e) other types of therapies, (f) general stress-focused therapy *via* Employee Assistance Program; and (g) no further recommendations. A combination of referrals was possible for any given patient.

#### Step 2 Measures

##### Traumatic Stress Symptoms

The PTSD Checklist for DSM-5 (PCL-5; Weathers et al., 2012) contains 20 self-report items answered on a 5-point Likert scale (1 = not at all to 5 = extremely) measuring probable stress-related disorders. Items were summed with a total score of 33 or above used to indicate risk (44). Participants were not required to endorse a Criterion A trigger prior to completing the PCL-5. Internal consistency was high for this sample (Chronbach's α = 0.95).

##### Depression

The Patient Health Questionnaire-9 [PHQ-9; (50)] is a 9-item self-report instrument answered on a 4-point Likert scale (0 = not at all to 3 = nearly everyday) for depression. A total score of 10 corresponding to “moderate” depressive symptoms was used to indicate risk. Internal consistency was high for this sample (Chronbach's α = 0.90).

## Results

### Step 1

At Step 1, ~43% of respondents screened positive for at least one identified risk (72 participants screened positive for one risk; 15 participants, two risks; eight participants, three risks; four participants, four risks). See Table 1. Probable diagnostic risk rates were shown at the following distributions: PTSD (7.9%), depression (9.6%), anxiety (13.5%), and problematic alcohol use (36.7%), see Table 2. Individuals who screened positive for any risk (*n* = 99) were referred to Step 2.

**Table 1:** Number of identified risks at step 1.

**Number of identified risks**	**Percentage**
1 identified risk	72.7% (72)
2 identified risks	15.2% (15)
3 identified risks	8.1% (8)
4 identified risks	4.0% (4)

**Table 2:** Probable diagnostic rates at step 1.

**Diagnostic risk**	**Percentage**
PTSD	7.9% (18)
Depression	9.6% (22)
Anxiety	13.5% (31)
Problematic alcohol use	36.7% (84)

### Step 2

Of the 99 who screened positive and with attempts to contact them, 56 engaged in the virtual telehealth appointment. Among the 56 individuals who participated in Step 2, 17.9% were above the cutoff for probable stress-related disorders while 25% were identified as having moderate to severe levels of depression, see Table 3. Of the 56 who participated in Step 2, 67.86% (*n* = 38) were recommended to pursue additional mental health services beyond Step 2, which is equivalent to ~17% of full-time personnel who engaged in this intervention (denominator = 229). See Table 4 for the percentage of referrals by the level of care. Among the 38 individuals who participated in Step 2 and received a recommendation for follow up 76.32% (*n* = 29) adhered to these recommendations.

**Table 3:** Probably diagnotic rates at step 2.

**Diagnostic risk**	**Percentage**
PTSD	25.0% (14)
Depression	17.9% (10)

**Table 4:** Recommendations and follow up.

**Recommendation**	**Percentage**
Occupational trauma therapy only	25% (14)
Step 3 brain health screen	5.36% (3)
Health behavioral intervention	3.57% (2)
Other therapy	3.57% (2)
Maintain employee assistance program	1.79% (1)
Combination of referrals (e.g., occupational trauma therapy and step 3)	42.1% (16)

## Discussion

Ample evidence suggests that first responders are difficult to engage in mental health resources. Despite longstanding knowledge of mental health risks are incurred among firefighters as an occupational hazard, there is limited applied research and behavior service delivery that focuses on improving access and utilizization (27, 51). The goal of the present study was to describe an applied intervention aimed at decreasing barriers to access and promoting the utilization of services among firefighters.

In summary, we utilized a combined approach, applying barriers and facilitators identified by Jones et al. (27) into an intervention, thereby extending a Planned-Action framework. Specifically, Jones and colleagues identified these prime facilitators of mental health service utilization among first responders: increasing knowledge, reducing stigma, increasing positive experience with mental health providers, and removing time-based burdens. We intentionally targeted each of these factors (e.g., flexible scheduling using a telehealth platform; stigma reduction using an “opt-out” strategy and a normalizing “performance enhancement” focus [rather than a pathology orientation]; increase positive experience by providing cultural training for providers). Additionally, we implemented the intervention using a cost-conscious and utilization-boosting stepped care implementation design [see (52)].

Our findings show that the current intervention was engaged by ~56% of “at-risk firefighters.” More than half of those who screened positive and engaged in a telehealth visit were provided with recommendations for further mental health service follow-up (*n* = 38), among whom 76% (*n* = 29) followed through with these referrals. This is comparable to 62% recommendation adherence in other samples [e.g., (52)]. Our high level of adherence to follow-up recommendations suggests that low-intensity intervention such as the one presented is useful in promoting needed, preventative mental health service use among firefighters.

First responders are often thought of as individuals who move toward crises to come to the aid of others. This intervention mirrored that sentiment by “moving toward” first responders and meeting them where they are. The clinical scheduling assistant reached out to participants within 48–72 h after screening positive for risk. Prioritizing individualized communication likely increased participation with the stepped care intervention. Step 2 appointments were provided virtually, making it more feasible for participants to attend. It has been well-noted that aside from stigma-related barriers, structural barriers (53) often hinder first responders from engaging with mental health professionals. It is possible that follow through with the Step 2 session was boosted by the easily accessible format in this stepped care intervention.

It is also important to note that the number of firefighters who were referred for more than one clinical session was only ~17% of the total number who participated in this intervention (denominator = 229). This finding indicates the preliminary value in an approach such as ours for “right sizing” resource appropriations needed to provide substantive mental health services, although much work is yet to be done to further elucidate that cost/funding need across time (and to generalize beyond one fire department).

This study was limited by several factors. First, this intervention was implemented in a single fire department thus limiting the generalizability of findings to other departments. Further research needs to take this intervention system to multiple fire departments to increase power as well as account for potential differences in various stations. Second, this project addressed firefighters but no other first responder populations such as police officers, emergency medical technicians (EMTs), and emergency dispatch. To evaluate its effectiveness in other first responder fields this system needs to be tested in a variety of emergency service stations as well as among adequate numbers of first responders. Third, insufficient sleep was not measured in this sample in the Step 1 screening tool. It has been widely established that sleep problems are common among emergency personnel (54) due to several factors such as disruptions of circadian rhythm and exposure to occupational trauma (55). Examination of insufficient sleep should perhaps be included in any initiation of stepped care services, thus increasing opportunities to provide psychoeducation as well as individualized referrals to improve sleep. Fourth, funding constraints limited our ability to engage participants in qualitative interviews about their experience with the intervention. This is a critically missing component for future research to endeavor to maximally shape interventions to be effective across cultures. Fifth, some individuals who screened as “at-risk” chose not to participate in Step 2, and data on discontinuation was not gathered, such as reasons for discontinuing despite being identified with a mental health service need is an important next step. Future studies should aim to examine the reasons for declining participation to better serve this population. Sixth, we do not have comprehensive follow-up data on either the efficacy of the brief problem-focused interventions administered or the longer-term efficacy of engaging in this intervention. It is important for future studies with sufficient funding to pursue the value of such brief intervention approaches. Established dissemination and implementation frameworks [e.g., the Planned-Action framework; (29); see (30)] are built to provide a rigorous and larger-scale study of interventions such as this. Such work would help to improve and expand these promising preliminary findings, to promote high quality and best practices to help draw in the largest proportion of “in need” first responders.

While the research on mental health outcomes for first responders is limited, what does exist indicates that this group is at significant risk for psychiatric conditions and that they are hesitant to seek help. However, such hesitancy to seek help, whether it be due to stigma or barriers to access, should not be equated with disinterest in receiving help when needed or offered. The results of this project are encouraging as it shows that by providing appropriate and tailored levels of care, firefighters can be open to receiving mental health referrals and will engage with said referrals. The work of a first responder has always presented numerous challenges that increase the risk of psychopathology. But with the ever-increasing number of natural disasters, social unrest, and the ongoing COVID 19 pandemic, finding ways to increase access to mental health resources and decrease the stigma among those services is more important than ever. Much work remains to be done to evaluate the effectiveness of mental health treatment in this population.

## Data Availability Statement

The data presented in this article is not readily available given the nature of this research, participants of this study did not agree for their data to be shared publicly. Requests to access the data should be directed to aj.smith-2@dartmouth.edu.

## Ethics Statement

The studies involving human participants were reviewed and approved by University of Utah IRB. The patients/participants provided their written informed consent to participate in this study.

## Author Contributions

HW and DFH wrote the manuscript. All authors contributed to the article and approved the submitted version.

## Funding

Program Description Issued By US Department of Homeland Security (DHS), Federal Emergency Management Agency (FEMA)/Grant Programs Directorate (GPD), Catalog of Federal Domestic Assistance (CFDA) Number 97.044, CFDA Title Assistance to Firefighters Grants (AFG), Notice of Funding Opportunity Title FY 2018, Assistance to Firefighters Grants, Notice of Funding Opportunity Number DHS-18-GPD-044-00-99, Authorizing Authority for Program Section 33 of the Federal Fire Prevention and Control Act of 1974, Pub. L. No. 93-498, as amended (15 U.S.C § 2229), Appropriation Authority for Program Department of Homeland Security Appropriations Act, 2018 (Pub. L. No. 115-141), and Program Type New Grant ID: EMW-2018-FO-05385.

## Conflict of Interest

The authors declare that the research was conducted in the absence of any commercial or financial relationships that could be construed as a potential conflict of interest.

## Publisher's Note

All claims expressed in this article are solely those of the authors and do not necessarily represent those of their affiliated organizations, or those of the publisher, the editors and the reviewers. Any product that may be evaluated in this article, or claim that may be made by its manufacturer, is not guaranteed or endorsed by the publisher.
